# Vaping and COVID-19: Insights for Public Health and Clinical Care from Twitter

**DOI:** 10.3390/ijerph182111231

**Published:** 2021-10-26

**Authors:** Anuja Majmundar, Jon-Patrick Allem, Jennifer B. Unger, Tess Boley Cruz

**Affiliations:** Department of Preventive Medicine, Keck School of Medicine, University of Southern California, Los Angeles, CA 90032, USA; allem@usc.edu (J.-P.A.); unger@usc.edu (J.B.U.); tesscruz@usc.edu (T.B.C.)

**Keywords:** vaping, COVID-19, Twitter, temporal trends, health education, public health

## Abstract

This study describes key topics of discussions on Twitter at the intersection of vaping and COVID-19 and documents public reactions to announcements from authoritative health agencies. Twitter posts containing vaping and COVID-19-related terms were collected from 1 December 2019 to 3 May 2020 (*n* = 23,103 posts). Text classifiers and unsupervised machine learning were used to identify topics in posts. Predominant topics included *COVID-19 Respiratory Health* (18.87%), *COVID-19 Susceptibility* (17.53%), *Death* (10.07%), *Other COVID-19 Health Effects* (9.62%), and *Severity of COVID-19* (7.72%), among others. Public conversations on topics, such as *Severity of COVID-19*, *Transmission*, *Susceptibility*, *Health Effects*, *Death*, and *Smoking cessation*, were shaped by announcements from U.S. and international health agencies. Armed with the insights from this study, medical providers should be prepared to discuss vaping-related health risks with their patients in the era of COVID-19. Misconceptions around vaping as a protective behavior from, and an effective treatment against, COVID-19 should also be corrected.

## 1. Introduction

The United States is currently experiencing a vaping epidemic and a COVID-19 pandemic. Juxtaposing scientific arguments about the role of vaping in COVID-19-related risks and symptoms may create widespread concerns and misconceptions among the public. Scientists hypothesize that vaping, associated with acute pulmonary and immunologic toxicity, may increase susceptibility to COVID-19 infections [1]. In contrast, other arguments suggest investigating nicotine’s role as a therapeutic option for COVID-19 infections [2]. It is, therefore, crucial to understand how the public contextualizes the role of vaping with COVID-19-related risks and symptoms. Medical providers and public health professionals can use this information to understand misconceptions and, eventually, tailor communication campaigns to address them.

Twitter is a popular social media platform comprised of organic public discussions on timely topics. Approximately one-quarter of U.S. adults and 42% of U.S. young adults aged 18–29 years old report using Twitter [3]. Previous research on Twitter content has provided useful insights about the public’s beliefs about, attitudes toward, and experiences surrounding health behaviors and disease [4]. Twitter surveillance of public conversations, including on vaping, has also highlighted the impact of state-level, national-level, and international public health agencies on social media discourse [5]. The present study describes key topics of discussions on Twitter at the intersection of vaping and COVID-19 and documents public reactions to announcements from authoritative health agencies to inform future risk communication efforts, providing reliable updates, encouraging positive health behaviors, and addressing misconceptions.

## 2. Methods and Materials

Twitter posts containing both vaping-related terms and COVID-19-related terms were obtained from 1 December 2019 to 5 May 2020 using Twitter’s streaming application program interface (API) while specifying that the data be limited to posts originating from the United States. Please refer to the Appendix A.1 and Appendix A.2 for the list of vaping- and COVID-19-related keywords. The initial corpus consisted of 112,432 posts originating from the United States of America (USA). Similar to prior research [6], retweets, non-English posts, posts from social bots [7], duplicate posts, spam, and promotional posts were excluded from the sample. To identify social bots, Python script was used to run each username in the analytic dataset through Botometer. The analytic sample consisted of 23,103 posts from 13,823 unique accounts.

To synthesize pertinent information, we performed basic normalization (e.g., lower case all text, remove special characters), lemmatization (e.g., “vaper”, “vaper’s”, and “vapers’” were all converted to “vaper”), stop word removal using the NLTK library in Python 3.0 removal (e.g., words such as “the” and “of”) and frequently occurring search terms, non-printable character removal (e.g., emojis and symbols from other languages), and the normalization of Twitter user mentions (e.g., “@jimjohn” was converted to “@Person”).

To identify key topics of conversation, posts were analyzed using word frequencies of single words (one-grams) and double-word (bi-gram) combinations. For example, a normalized tweet ‘she love vape’ consists of 3 one-grams (e.g., she, love, vape) and 2 bi-grams (e.g., she–love, love–vape). One-grams and bi-grams were then visualized using word clouds for further inspection of emerging topics. Based on wordcounts and visual assessments, the authors identified an initial list of topics.

Next, GloVe [8], an unsupervised learning algorithm developed by the Stanford NLP group, was used to expand the above list of one-grams and bi-grams by identifying words similar to the one-grams and bi-grams identified for each initial topic. GloVe (2B tweets, 27B tokens, 1.2M vocab, uncased, 200d vectors) was retrained on the study sample to learn COVID-19- and vaping-related vocabulary. GloVe includes pre-trained vector representations for words. It calculates the Euclidean distance (or cosine similarity) between two-word vectors to measure the linguistic or semantic similarity of the corresponding words (e.g., ‘kale’ and ‘spinach’). The vector difference between two-word vectors captures the meaning specified by the juxtaposition of two words. This method enabled us to identify colloquial terms (e.g., ‘juuling’ is often used to refer to vaping) used in some contexts to expand the list of words used to identify topics.

Final classification of each post to one or more topics was implemented by using rule-based classifiers that checked for the presence of topic-specific one-grams and bi-grams in a tweet. Additionally, posts could be classified to more than one topic. For instance, the post *“If you got fever from COVID, chances are that it spread from your vaping friends to you.”* would be categorized under *COVID-19 Transmission* and *Other COVID-19 Health Effects*. A total of 2000 randomly selected tweets from the analytic sample were manually reviewed to ensure rule-based classification of tweets to topics was appropriately carried out (i.e., posts were reflective of the topic). All tweets were determined to be appropriately classified. Please refer to Table 1 for definitions and example Twitter posts of predominant COVID-19-related topics.

**Figure 1:**
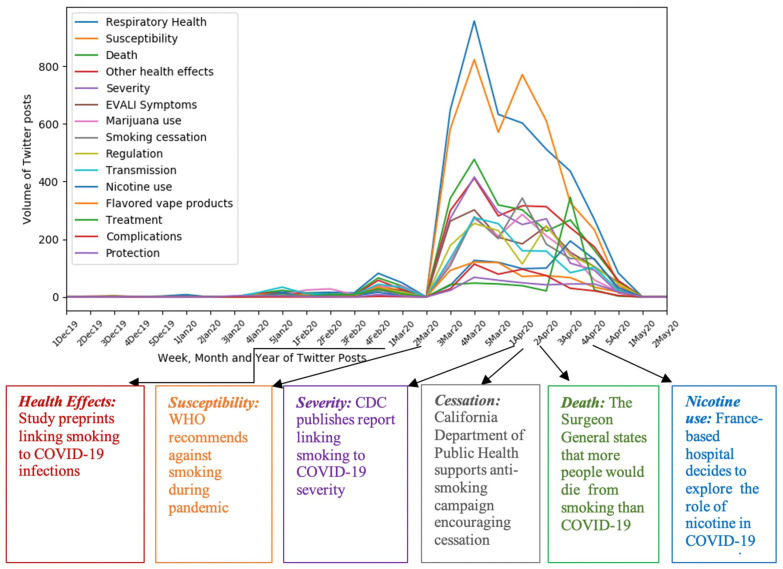
Temporal patterns of topics in the context of announcements from authoritative public health agencies.

We also documented public reactions to announcements from authoritative health agencies during the study period. To accomplish this objective, we first plotted the weekly temporal patterns of volume of posts associated with each topic identified in the data (Figure 1) and then retrospectively reviewed the public health-related announcements from the agencies to identify overlaps in the timelines of the announcements and topics of conversations.

All analyses relied on public, anonymized data; adhered to the terms and conditions, terms of use, and privacy policies of Twitter; and were performed under Institutional Review Board approval from the authors’ university. To protect privacy, no tweets were reported verbatim in this paper.

## 3. Results

The total coverage of the 15 identified topics constituted 62.10% of all tweets in the sample. The remaining tweets were too varied to be classified into a single topic with meaningful coverage (i.e., less than 1% of total tweets in the sample). Predominant topics included *COVID-19 Respiratory Health* (18.87%), *COVID-19 Susceptibility* (17.53%), and *Death* (10.07%). Additional topics included *Other COVID-19 Health Effects* (9.62%), *Severity of COVID-19* (7.72%), *EVALI (E-cigarette or Vaping Use-Associated Lung Injury) Symptoms* (6.94%), *Marijuana use* (6.41%), *Smoking cessation* (6.34%), *Regulation* (6.08%), and *COVID-19 Transmission* (5.81%). Please see Table 1 for more details.

Figure 1 offers an overview of temporal patterns of all topics in response to announcements from authoritative public health agencies. The volume of Twitter posts associated with topics pertaining to *Respiratory Health*, *Death*, and *Other COVID-19 Health Effects*, and *COVID-19 Susceptibility* started to increase after news reports published in the first week of March 2020 of scientific findings linking smoking to COVID-19 infections [9,10], and the WHO’s recommendation on smoking abstinence during the pandemic (second week of March, 2020) [11]. *COVID-19 Severity* peaked right after the issue of the CDC’s report in the first week of April 2020, highlighting that patients who smoke might be at higher risk of severe COVID-19 infections than those who do not smoke [12] The topic of *Smoking cessation* peaked after the California Department of Public Health released its ongoing anti-smoking campaign, “No Butts” [13]. *Death* and *Nicotine use* peaked for the second time after the respective announcements from the Surgeon General’s statement highlighting the fact that smoking-related deaths would exceed COVID-19-related deaths in the second week of April 2020 [14] and a France-based hospital’s announcement to test the role of nicotine in COVID-19 disease progression in the last week of April 2020 [15].

## 4. Discussion

This study documented public conversations at the intersection of two salient public health issues: the ongoing COVID-19 pandemic and the vaping epidemic. Predominant topics of conversation pertained to *COVID-19 Respiratory Health*, *COVID-19 Susceptibility*, *Death*, *Other COVID-19 Health Effects*, *COVID-19 Severity*, *EVALI Symptoms*, *Marijuana use*, *Smoking cessation*, and *Regulation.* Temporal patterns of topic-related conversations in relation to health agency-related announcements showed the ebb and flow of public discussions. Armed with the insights from this study, medical providers should be prepared to discuss the risks involved in vaping with their patients in the era of COVID-19, including respiratory and other health effects, potential for increased susceptibility, complications, and severity of symptoms. While the scientific evidence on vaping-attributable COVID-19 health risks remains inconclusive [16] clinicians may be tasked with communicating medical uncertainties with their patients, i.e., translating emerging scientific findings to their patients and offering recommendations while acknowledging patients’ individual experiences.

Many posts found in this study discussed misconceptions about the protective effects of vaping in COVID-19 and the potential contribution of vaping to COVID-19 treatment. Prior research suggests that medical misinformation related to COVID-19 was regularly propagated on Twitter [17] Misconceptions held by patients could be countered by clinicians with two-sided statements that provide a brief acknowledgement of the misconception, then a refutation of the misconception, followed by a stronger argument supported by known scientific evidence. Misconceptions could also be addressed by tailored health communication campaigns.

Perceived similarities between EVALI and COVID-19-related symptoms were often discussed on Twitter, previously documented [18] but not systematically analyzed. Such similarities were also recently noted by the CDC in their analysis of eight patients in the state of California, who presented with EVALI symptoms but were later diagnosed with COVID-19 [19]. Clinicians may want to prepare themselves to address this confusion by translating medical information into lay terms to help patients distinguish between EVALI and COVID-19 disease processes.

*Death* was one of the predominant topics. Prior work investigating COVID-19-related Twitter posts originating from e-cigarette users and non-users suggests that *Death* was a predominant concern among e-cigarette users [20]. The topic of *Transmission* of COVID-19 included mentions of shared vaping devices, and health communication efforts, rating daily activities on a risk continuum (high–low risk) of COVID-19 transmission [21] may consider addressing this issue. Topics pertaining to marijuana and nicotine use were also found in this study and may be driven by self-isolation for community-level containment of the disease. For instance, research suggests that social distancing and self-isolation may impact substance use behaviors [1]. This may include substance use initiation among those isolated and stressed, and substance use relapse among those in recovery. Findings from this study may be an early warning sign of emerging risk behaviors pertaining to abuse liability and addiction during this pandemic.

Many topics peaked briefly in response to public health organizations’ announcements. Previous work suggests that solution-based risk communication, incorporation of visual imagery in posts, and acknowledgment of public concerns from well-known health agencies are highly engaging on social media platforms [22]. Public health agencies may consider these strategies for prolonged engagement with the public on social media.

The ability to identify distinct weekly temporal patterns of topics of conversation is a strength of using Twitter in public health surveillance. Temporal analyses from this study suggested that topics of conversation, such as COVID-19 severity, transmission, susceptibility, health effects, death, and smoking cessation, were shaped by announcements from national and international health agencies. However, topics increased only briefly. For sustained public engagement on social media platforms, public health agencies may consider incorporating images with posts and acknowledging public concerns from well-known health agencies [22].

The findings from this study should be considered with several limitations in mind. For instance, this study focused on posts to Twitter and findings may not generalize to other social media platforms. The posts in this study were collected from a 5-month period and may not extend to other time periods. Data collection relied on Twitter’s streaming API, which prevented the collection of posts from private accounts. Additionally, the temporal analysis does not imply causation and may be confounded with other COVID-19-related discussions, news, and policy changes (e.g., shelter in place orders across the U.S.). Twitter does not reflect the attitudes and behaviors of the entire U.S. public. It should also be noted that topics of conversation were informed by GloVe-word embedding algorithms, which may group keywords together that are statistically independent of each other. While our analysis reveals potential misconceptions related to COVID-19 and vaping, it did not quantify topics in terms of the volume of posts pertaining to potential misconceptions. While our analysis identified 15 topics from the data, we were unable to test if this 15-topic model was optimal. That is, given our mixed methods approach, involving manually reading posts in their entirety and unsupervised machine learning, we were unable to provide a goodness of fit measure or coherence score that are common in studies that rely on machine learning alone.

Despite these limitations, this study suggests that Twitter can inform education efforts around COVID-19 by highlighting public concerns and potential misconceptions. Future work may consider dissecting public misconceptions on Twitter, such as posts highlighting protective health effects of vaping from COVID-19, the role of vaping in COVID-19 treatment, and the valence of topics for more nuanced insights. Future work may also consider characterizing topics signaling emerging public misperceptions, such as posts highlighting protective health effects of vaping in COVID-19 and the role of vaping in COVID-19 treatment by source (e.g., individuals and interest groups), to identify targets for future interventions preventing risky behaviors informed by social media misinformation. Continued surveillance of patterns of risky health behaviors, such as nicotine and marijuana use during the COVID-19 pandemic, may also play a vital role in informing future health interventions.

## Figures and Tables

**Table 1:** Predominant topics referencing COVID-19 and vaping on Twitter.

Topic Label	Definition	Paraphrased Posts	*n*	%
COVID-19 Respiratory Health	Posts discussed COVID-19 respiratory problems and concerns due to vaping	*Vaping perhaps causes pulmonary inflammation. Perfect recipe for covid19 infection.* *My parents freak out cause I vape cause there is a connection between COVID and vaping and how vaping weakens your respiratory system.*	4359	18.87
COVID-19 Susceptibility	Posts discussed fear or risk of contracting COVID-19 as a result of vaping	*Those who vape are more at likely to get coronavirus.* *Because they vape nicotine makes them 5x to 10x less likely to catch the covid disease.*	4049	17.53
Death	Posts discussed concerns about dying from COVID-19 and vaping	*Guys, go ahead and continue vaping if you want to die from COVID-19.* *You’ll get yourself and us killed from coronavirus if you keep vaping.*	2326	10.07
Other COVID-19 Health Effects	Posts discussed other COVID-19 health effects besides respiratory and death-related outcomes as a result of vaping	*I have chest pain–should I blame vaping or covid?* *I had flu-like symptoms from covid19 and my mom thinks it’s because of vaping.*	2222	9.62
COVID-19 Severity	Posts discussed the degree of severity of COVID-19 symptoms among those who vape	*Do you all realize that vaping causes more severe COVID-19?* *Scientists see vaping as a possible risk factor for severe covid19 coronavirus infection*	1784	7.72
EVALI Symptoms	Posts discussed the similarity of symptoms related to COVID-19 and EVALI (E-cigarette, or vaping, product use-associated lung injury) and potential misdiagnosis	*We dropped the ball on a hoax because COVID-19 might have been EVALI.* *The death rate of EVALI is 44 CVOID19 is 5 at first and down to 2. Now I am pretty sure for the EVALI cases they did not check the virus DNA assay.*	1603	6.94
Marijuana Use	Posts discussed use or purchase of marijuana and/or cannabis during the COVID-19 pandemic	*I have a sore throat from being at home weed vaping during covid. I guess I it a bit too much.* *So you allow alcohol shops to remain during covid but not weed or vape shops?*	1481	6.41
Smoking Cessation	Posts discussed challenges and opportunities in smoking cessation due to lockdowns during the COVID-19 pandemic	*All vape shops are shutdown. How am I expected to quit smoking during covid19 lockdown?* *Now is the time to quit smoking and switch to vaping! #covid*	1465	6.34
Regulation	Posts discussed the role of, or mentions of, regulatory agencies, such as the FDA or CDC, during the COVID-19 pandemic.	*The FDA will bankrupt thousands of vape shops inspite of this coronavirus pandemic.* *CDC hasn’t found one case of a vaper having covid so far.*	1404	6.08
COVID-19 Transmission	Posts discussed ways people believe the COVID-19 virus is transmitted as a result of vaping	*Is it possible that COVID can spread from person to person by vaping products?* *Can covid19 spread through secondhand smoke if an individual is close to a person smoking?*	1343	5.81
Nicotine Use	Posts discussed use of, or the role of, nicotine-containing substances during the COVID-19 pandemic.	*How do I get my fill of nicotine when everything is shutdown? #covid.* *Does anyone know if vaping nicotine really decreases chances of getting covid?*	898	3.89
Flavored Vape Products	Posts discussed flavored vape product use and bans in the COVID-19 pandemic.	*If you could have the same compassion for us as you would for someone with covid, please allow us to still purchase flavors and products online.* *You guys I know lots of people are sick and dying right now but do not worry some folks have the solution: banning flavored vapes during covid19.*	662	2.87
COVID-19 Treatment	Posts discussed how vaping could aid in the treatment of COVID-19	*Are you guys aware that vaping vaping can treat all symptoms of COVID-19?* *Vape pens could directly deliver covid meds to the lung for treatment.*	636	2.75
COVID-19 Complications	Posts discussed COVID-19-related complications among those who vape	*Vaping can worsen infections from COVID-19.* *These youngsters who are now having complications from covid19 use vape pens the most.*	450	1.95
Protection	Posts discussed potential protective effects of vaping in COVID-19	*Propylene glycol found in vaping liquids has shown to have protected people from COVID-19.* *The e-liquid and vape salts have antiviral properties that can prevent covid.*	375	1.62

## Data Availability

Available upon request.

## Appendix A

### Appendix A.1. COVID-19-Related Search Terms

‘chinesevirus’, ‘covidiot’, ‘covid’, ‘covid2019’, ‘2019ncov’, ‘codvid_19’, ‘codvid-19’, ‘covid-19’, ‘codvid19’, ‘coronavid19’, ‘covd19’, ‘covid_19’, ‘covid19’, ‘briefing_covid19’, ‘ncov’, ‘ncov2019’, ‘convid19’, ‘corvid19virus’, ‘coronaapocolypse’, ‘coronapocalypse’, ‘caronavirusoutbreak’, ‘coronaoutbreak’, ‘caronavirus’, ‘viruscorona’, ‘coronavirususa’, ‘conronaviruspandemic’, ‘coronaviruspandemic’, ‘coronavirus’, ‘coronaflu’, ‘coronapandemic’, ‘coronapanik’, ‘coronavirustruth’, ‘coronavirusupdate’, ‘infocoronavirus’, ‘novelcorona’, ‘novelcoronavirus’, ‘coronaalert’

Note: These search terms were indentified from Twitter’s official list of COVID-19 search terms as of 11 March 2020 (Link: https://developer.twitter.com/en/docs/labs/covid19-stream/filtering-rules, accessed on: 3 November 2020). Twitter, Inc. updated the page with additional keywords since then.

### Appendix A.2. Vaping-Related Search Terms

‘ecig’, ‘ecigs’, ‘ecigarette’, ‘ecigarettes’, ‘e-cigarette’, ‘e-cigarettes’, ‘e-liquid’, ‘e-liquids’, ‘eliquid’, ‘eliquids’, ‘ejuice’, ‘ejuices’, ‘e-juice’, ‘e-juices’, ‘vaping’, ‘vapes’, ‘vape’, ‘vaper’, ‘juul’, ‘juuling’.
